# Viruses with different genome types adopt a similar strategy to pack nucleic acids based on positively charged protein domains

**DOI:** 10.1038/s41598-020-62328-w

**Published:** 2020-03-25

**Authors:** Rodrigo D. Requião, Rodolfo L. Carneiro, Mariana Hoyer Moreira, Marcelo Ribeiro-Alves, Silvana Rossetto, Fernando L. Palhano, Tatiana Domitrovic

**Affiliations:** 1 Universidade Federal do Rio de Janeiro, Instituto de Bioquímica Médica Leopoldo de Meis, Rio de Janeiro, 21941-902 Brazil; 2 Fundação Oswaldo Cruz, Instituto Nacional de Infectologia Evandro Chagas, Rio de Janeiro, 21040-900 Brazil; 3 Universidade Federal do Rio de Janeiro, Instituto de Matemática, Rio de Janeiro, 21941-902 Brazil; 4 Universidade Federal do Rio de Janeiro, Instituto de Microbiologia Paulo de Góes, Rio de Janeiro, 21941-902 Brazil

**Keywords:** Computational biology and bioinformatics, Virology

## Abstract

Capsid proteins often present a positively charged arginine-rich sequence at their terminal regions, which has a fundamental role in genome packaging and particle stability for some icosahedral viruses. These sequences show little to no conservation and are structurally dynamic such that they cannot be easily detected by common sequence or structure comparisons. As a result, the occurrence and distribution of positively charged domains across the viral universe are unknown. Based on the net charge calculation of discrete protein segments, we identified proteins containing amino acid stretches with a notably high net charge (*Q* > + 17), which are enriched in icosahedral viruses with a distinctive bias towards arginine over lysine. We used viral particle structural data to calculate the total electrostatic charge derived from the most positively charged protein segment of capsid proteins and correlated these values with genome charges arising from the phosphates of each nucleotide. We obtained a positive correlation (r = 0.91, p-value <0001) for a group of 17 viral families, corresponding to 40% of all families with icosahedral structures described to date. These data indicated that unrelated viruses with diverse genome types adopt a common underlying mechanism for capsid assembly based on R-arms.

## Introduction

The most common solution that viruses employ to protect their genomes is to assemble a spherical shell composed of multiple copies of only one or a few kinds of proteins. Capsid proteins (CP) interact with each other, usually following the principles of icosahedral symmetry, where the number of subunits forming the capsid is given by the triangulation number (T) × 60. The genome is either packaged during assembly driven by CP interactions (most eukaryotic viruses) or is pumped into a preformed capsid by a strong, virally encoded, ATP-dependent molecular motor (as with most bacteriophages)^1^. The second architecture is a helical arrangement of proteins (nucleocapsid proteins, NCP) that interact with the genome^2,3^. The mechanisms involved in the assembly of the protein shell and condensation of the viral capsid genome often find direct applications in the fields of drug development and nanotechnology.

Some icosahedral viruses have a high concentration of positively charged amino acid residues at the extremities of their CPs, known as arginine-rich motifs, poly-arginine, or arginine-arms (R-arms). These R-arms are directed towards the interior of the viral particle, where they can contact the encapsulated nucleic acid^4^. Studies with hepatitis B virus^5^, circovirus^6^, nodavirus^7^, and other models^8,9^ have demonstrated that these positively charged domains are essential for interaction with the viral genome and particle stability. Part of the functional explanation may be the counteraction of repulsive forces that results from the negatively charged nucleic acids condensed inside the capsid^10,11^. Different groups, working with single-stranded positive sense (+) RNA viruses, observed that the sum of net charges of all R-arm containing proteins in a virus capsid correlates with its genome packing capacity, e.g.^11–15^. However, for some specific viruses, R-arms have also been implicated in the interaction with cellular membranes promoting particle penetration into the cell^16^ or intracellular localization^17,18^. In these cases, R-arms can act as localization signals or cell-penetrating peptides^19,20^, suggesting that these domains are multifunctional.

Although R-arms are present in different viruses and are critical components for viral replication and assembly, they have never been formally annotated as a protein domain by widely known resources and databases, such as the Pfam protein family database^21^ or InterPro^22^. Consequently, there is no information on the distribution of R-arms across different organisms or viral families or their overall amino acid composition. This broad view perspective is necessary to determine if R-arms can be considered a typical functional module of icosahedral viral capsids and if they can be used to infer capsid assembly mechanisms.

R-arms often present low sequence conservation and extensive variation in length, which hampers domain identification by profile Hidden Markov-Model (HMM) protein classification, the method employed by protein databases as Pfam^21^. Moreover, R-arms often lie within an intrinsically disordered region that is too dynamic or flexible to be resolved in viral capsid structural models generated by X-ray crystallography or cryo-electron microscopy. These attributes complicate the use of traditional approaches for the identification of R-arms in unrelated viruses and sometimes even within a viral family.

In this study, to determine the occurrence of positively charged domains among proteins from different viruses, we analyzed the net charge distribution across the primary structure of proteins deposited in the reviewed Swiss-Prot database. Using a program that calculates the net charge in consecutive amino acid stretches, we observed that icosahedral viruses are enriched with positively charged stretches, similar to other nucleic acid binding proteins, especially at extreme charge values (≥+17). The viral capsid segments also present at least four times more arginine than lysine, a feature that is not common in cellular proteins. We also made a focused effort to calculate the correlation between the total net charge derived from the positively charged domain and the genome charge for a comprehensive group of viruses with different genome types. We demonstrate that the capsid net charge is closely related to genome size in most icosahedral viruses, independent of genome type, and that highly charged domains are a strategy employed by some viruses to package relatively large genomes. We propose that this analysis can be used to predict whether the electrostatic interaction between the positively charged domain and the genome is an important driving force for capsid assembly and stability.

## Results

### Viral proteomes are enriched with super-positive stretches

The first step to characterize the charge distribution along different protein sequences was to define the length of the search frame, that is, the number of residues that would be used for net-charge calculation in every consecutive stretch. Positively charged motifs of viral proteins can be in rigid patches on the inner capsid surface (e.g., bacteriophage MS2, *Leviviridae*^23^), but usually, they are within helical or flexible arms in the N-terminus (Fig. S1). Therefore, a commonly used criterion for R-arm size determination is the length of the disordered region of the N-terminus as determined by x-ray crystal models^12^ or secondary structure prediction software^24^. We listed (+)RNA viruses that have been previously analyzed^11,15,24^ and noticed that the average unstructured N-terminus is approximately 30 amino acid residues (n = 14 families, SD ± 23.71). Even though R-arms are not necessarily restricted to disordered regions, these observations indicated that this frame size was a good starting point for our analysis.

**Figure 1:**
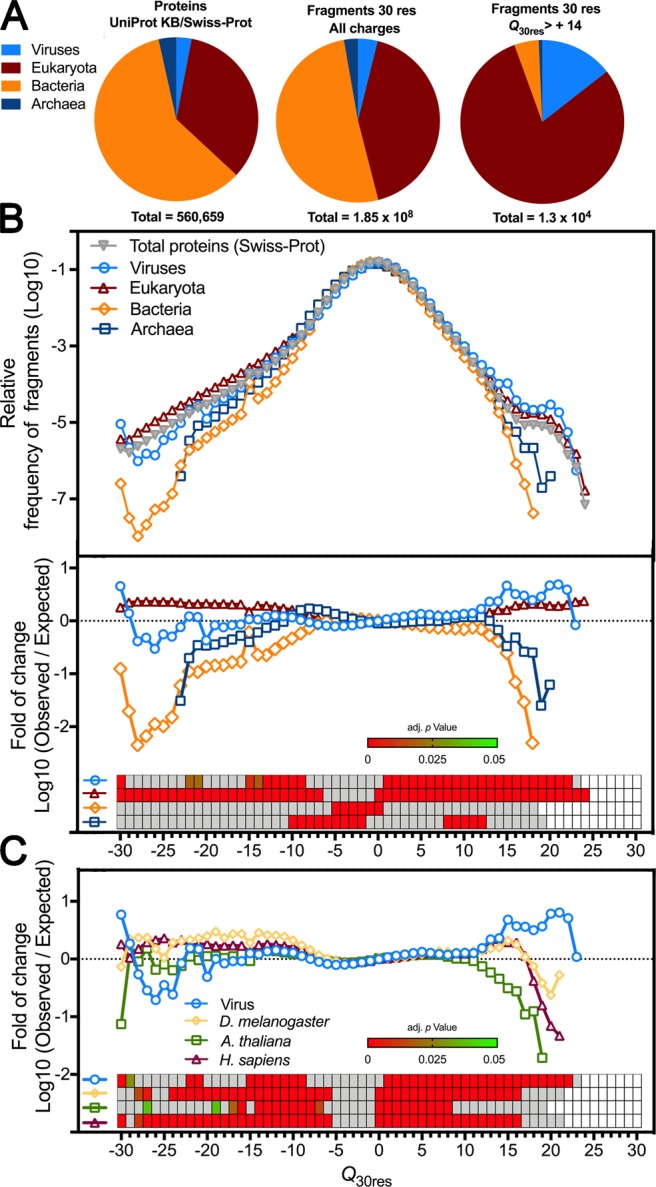
Viral proteins are enriched with positively charged stretches. Protein sequences derived from the reviewed Swiss-Prot data-bank (560,659 proteins) were used as input for a program that calculates the net charge of every consecutive 30 amino acid residues (Q_30res_). (**A**) Numerical proportion of the protein entries and the calculated 30 residues fragments from the 4 domains of life: viruses (16,866 proteins, 7.2 × 10^6^ fragments), Eukaryota (190,054 proteins, 7.8 × 10^7^ fragments), Bacteria (334,178 proteins, 9.4 × 10^7^ fragments), and Archaea (19,561 proteins, 5.1 × 10^6^ fragments). (**B**) The upper panel shows the normalized net-charge frequency distribution of protein segments from the four domains of life, and the lower panel shows the observed vs. expected net-charge frequency of each protein group shown in B in relation to the total Swiss-Prot proteome. The statistical enrichment analysis is shown in a heatmap (inset), where significant p-values are shown in shades of green to red. Grey slots represent p-values > 0.05. (**C**) Observed vs. expected net-charge frequency plot comparing viruses and the proteome of individual multicellular organisms: *Drosophila melanogaster* (3,279 proteins), *Arabidopsis thaliana* (14,430 proteins*)*, and *H. sapiens* (20,214 proteins).

To characterize the distribution of positively charged protein stretches in several organisms, we used a program that can screen a protein sequence and calculate the net charge every consecutive frame of 30 amino acid residues (e.g., 1–30, 2–31), generating a list of net-charge values Q_30res_^25^ (see Fig. S2 for more details). We analyzed the total UniProt KB/Swiss-Prot reviewed proteome (560,659 proteins, 1.85 × 10^8^ fragments) and separated the viral sequences from the three domains of life (Fig. 1A): Viruses (16,866 proteins, 7.2 × 10^6^ fragments), Eukaryota (190,054 proteins, 7.8 × 10^7^ fragments), Bacteria (334,178 proteins, 9.4 × 10^7^ fragments), and Archaea (19,561 proteins, 5.1 × 10^6^ fragments). Next, we generated a frequency distribution of the fragments according to their *Q*_30res_ values normalized by the total number of fragments in each group (Fig. 1B). We also calculated the fold of change between the observed *Q*_30res_ frequency values for a selected group of proteins (e.g., viral proteins) in relation to the expected frequency value calculated from the total proteome distribution (Fig. 1B, lower panel). We observed that even though eukaryotes contributed the majority of positively charged segments (Fig. 1A), viruses had the highest relative frequency and fold of enrichment values. When viruses were compared with protein groups from specific eukaryotes, such as *Drosophila melanogaster* (3,279 proteins), *Arabidopsis thaliana* (14,430 proteins), and *Homo sapiens* (20,214 proteins), viral proteins were the only class enriched in extremely high positively charged segments (charge ≥+17) (1C-inset with *p*-values).

**Figure 2:**
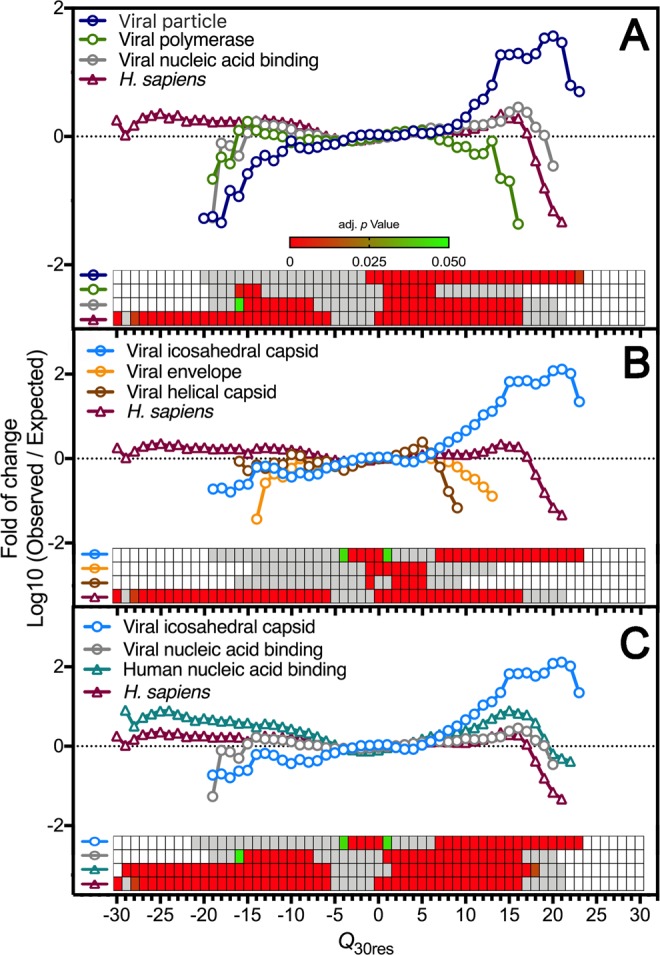
Capsid proteins from icosahedral viruses concentrate most of the positively charged protein segments of the viral proteome. Protein sequences derived from the reviewed Swiss-Prot data bank were used as input for a program that calculates the net charge of every consecutive 30 amino acid residues (Q_30res_). The observed vs. expected frequency of fragments net charge from a specific protein functional class in relation to the Swiss-Prot proteome. (**A**) The viral protein data set were divided into three different functional categories: viral polymerase (containing all different kinds of viral polymerases; 1,212 proteins); nucleic acid-binding (containing viral transcriptional/translational regulators, RNAi suppressors; 3,202 proteins); and viral particle (containing structural proteins present in viral particles; 1,902 proteins). (**B**) The viral particle data set was further divided into three different functional categories: Viral envelope (containing mainly glycoproteins; 808 proteins); Viral helical capsid (containing mainly nucleocapsid proteins from helical viruses; 232 proteins); and Viral icosahedral capsid (containing mainly capsid proteins from spherical viruses; 762 proteins). (**C**) The Viral icosahedral capsid dataset and the Viral nucleic acid binding dataset were compared to the Human nucleic acid binding data set (containing RNA and DNA binding proteins with diverse functional roles; 4,073 proteins). The statistical enrichment analysis is shown by a heatmap, where significant p-values are represented in shades of green to red. Grey slots represent p-values > 0.05.

Positively charged protein stretches can be involved in diverse roles, such as membrane interaction, DNA or RNA binding, and cellular localization signaling^26^. All these functions are important for virus replication and must contribute to the charge distribution profile of the viral protein dataset. To characterize the charge distribution according to protein function, we grouped viral proteins following their functional annotation available in the Swiss-Prot database (Fig. 2). As expected, proteins classified in the DNA/RNA binding functional class (i.e., viral transcriptional factors, RNAi suppressors) were more enriched in positively charged segments than the Total Swiss-Prot proteome (Fig. 2A). However, the “viral particle” subset had even higher frequencies of positively charged fragments (Fig. 2A). In Fig. 2B, we dissected the viral particle components and observed that the class containing the highest frequencies and broadest distribution of positively charged segments was “viral icosahedral capsid.” Even compared to human DNA/RNA binding proteins, viral icosahedral capsid proteins concentrated more positively charged segments than any other analyzed classes (Fig. 2C). The absolute distribution values of Figs. 1 and 2 can be found in Supplementary File S3.

### Positively charged domains of icosahedral capsids are mainly involved in capsid assembly and stability

We hypothesized that by searching for the most positively charged segment in a capsid protein (*Q*_max30res_) we could efficiently identify viral R-arm domains. Because the correlation between total R-arm charge and genome charge has already been demonstrated for a selected group of icosahedral RNA viruses^11,15,24^, we decided to generalize this calculation for all the icosahedral viruses in our dataset. This finding would not only validate our R-arm identification method for the previously analyzed (+)RNA viruses but would also reveal how the positively charged domain of viruses with different genome types relates to the capsid packaging capacity. While the theoretical determination of the genome charge (Q_*genome*_) is straightforward (each phosphodiester bond produces one negatively charged phosphate group), the calculation of the total capsid charge derived from R-arms is more complicated. We carefully curated our protein dataset to select entries that corresponded to viruses with known capsid structures and complete genome sequences. The total R-arm net charge was calculated by multiplying the *Q*_max30res_ found in a protein capsid by the number of subunits forming the capsid (Total *Q*_max30res_). We accounted for deviations in icosahedral symmetry by using the actual subunit copy number (e.g., *Papillomaviridae*: pseudo T = 7, with 72 pentamers of L1 and 72 copies of L2; *Geminiviridae*: formed by two fused T = 1 capsids totaling 110 subunits; *Picornaviridae*: pseudo T = 3, formed by 60 copies of up to 4 different proteins). We excluded viruses with complex multicomponent capsids and with uncertain protein copy numbers per particle. With these criteria, we eliminated complex icosahedral viruses, such as *Adenoviridae*, *Reoviridae*, *Herpesviridae*, and spherical viruses with nucleocapsid complexes (*Flaviviridae* and *Rubivirus*). For bacteriophage calculations, we used the final capsid protein sequence that results from proteolysis after maturation. We excluded scaffold proteins, the special vertex that connects with the packaging machinery, and other minor components of the capsid, such as decoration proteins. In the case of *Microviridae*, in addition to the major CP protein F, we included the J and H peptides. These proteins are highly positively charged and are present in the mature virion^27^.

**Figure 3:**
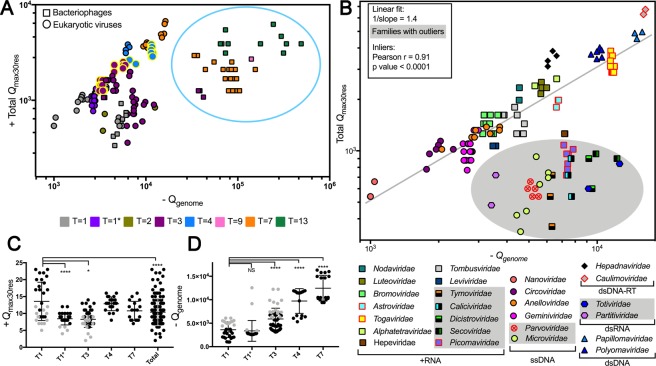
The entire capsid internal net charge calculated from the most positively charged capsid protein segment correlates with genome packing capacity. The maximum net-charge value found in a 30 amino acid residue stretch was multiplied by the number of subunits forming the capsid (Total Q_max30res_) of 179 viruses from 29 different families (see also S4). The total nucleic-acid net charge was calculated from the number of nucleotide residues in the genome (Q_genome_). For multipartite viruses, the longest genome segment was considered for the plot. (**A**) Scatter plot colored by T number. Circles and squares represent eukaryotic viruses and bacteriophages, respectively. The blue circle highlights the cluster formed by bacteriophages from the *Podoviridae*, *Siphoviridae*, and *Myoviridae* families. (**B**) Eukaryotic viruses and the bacteriophages *Leviviridae* and *Microviridae* were used to calculate a straight line fit (n = 133). The shaded area indicates families with outliers (ROUT 5%). Pearson correlation results obtained from the inliers (103) are shown in the inset. Data points contoured in red represent viruses that have more Lys than Arg in their positively charged segments (see also Fig. 5A). (**C**,**D**) Show the Q_max30res_ values per protein fragment and Q_genome_ values according to capsid T number, respectively. T1* corresponds to the T1 geminated capsids from *Geminiviridae* (110 subunits) and the dsRNA T1 capsids formed by dimeric subunits (120 subunits). Grey data points in (**C**,**D**) correspond to the outliers identified in panel A, as follows T1: ssDNA *Parvoviridae*; T1*: dsRNA *Totiviridae* and *Partitiviridae*; T3: all (+)RNA outliers *Caliciviridae*, *Dicistroviridae*, *Secoviridae*, *Picornaviridae*, and *Tymoviridae*. Error bars indicate the mean and SD values. Tukey’s p-values **** < 0.0001, *0.035.

The final list (S4) contained 179 icosahedral viruses from 29 different families and all genome types, except for single-stranded negative sense (−) RNA (all helical viruses) and ssRNA-RT, comprising 66% of virus families with known capsid structure (Viperdb). Figure 3A shows a scatter plot colored by T number; squares are bacteriophages, and circles are eukaryotic viruses. Symbols with yellow borders are +RNA viruses for which the positive correlation between R-arm charge and genome size was previously reported, thereby serving as a control for our analysis (*Alphatetraviridae*, *Nodaviridae*, *Togaviridae* and *Bromoviridae*).

We found a modest positive correlation between genome charge and total Q_max30res_ (Pearson r = 0.53, p-value <0.001) for the entire data set, including complex dsDNA bacteriophages. However, we observed that phages with ATP-dependent packaging molecular machines (*Siphoviridae*, *Myoviridae*, and *Podoviridae*) formed a cluster (blue circle), separated from small phages, *Leviviridae* (+RNA T = 3) and *Microviridae* (ssDNA T = 1) and other eukaryotic viruses. Hence, we excluded the big bacteriophages and analyzed the other viruses separately (Fig. 3B). A linear fit allowing outlier identification indicated that 20 viruses, members of 9 virus families (marked in grey), deviated from the fit (Fig. 3B).Assuming these families as outliers, we analyzed the remaining 103 inliers from 17 families, including the controls, in a correlation analysis. We obtained a Pearson (r) of 0.91 and a p-value <0.0001. This group (3B – inlier points) represents a subset of viruses for each genome packaging capacity is highly correlated to the internal net charge of the capsid. Figure 3, panels C and D show the *Q*_max30res_ and the *Q*_genome_ vs. T, respectively. Interestingly, T = 1 ssDNA viruses carried segments with the highest net charge (Fig. 3C), probably to maximize the packaging capacity of the smallest capsid of the viral world. Members from the *Circoviridae* family use only 60 subunits to pack genome sizes equivalent to the *Geminiviridae* (110 subunits) and *Bromoviridae* (180 subunits) (see Fig. 3B,C). We conclude that positively charged domains are a common strategy for capsid assembly and stabilization employed by viruses of different genome types and hosts. The clear separation of phages that use molecular motors to pump and concentrate the genomes under pressure into the capsid (blue group, Fig. 3A) supported that the plot can be used to infer if positively charged domains are involved in the genome packaging/capsid stability. The identification of outliers from the linear regression (Fig. 3B), that included control viruses, suggested alternative functions for these positively charged domains. The outlier position also indicates that these species have assembly strategies that are less dependent on electrostatic interactions between the genome and the capsid protein (see Discussion for more details).

**Figure 4:**
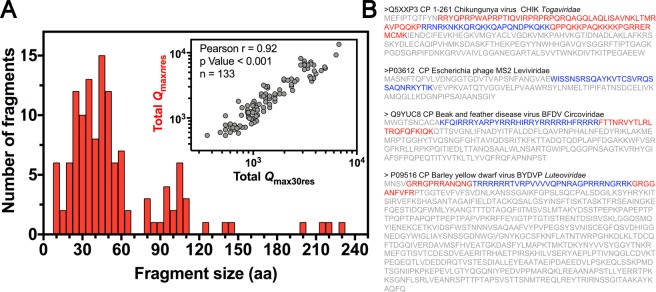
Automatic identification of positively charged domains with variable sizes. We designed and implemented an algorithm that calculates the net charge and the charge concentration *Q*c = *Q*/frame size) in incremental frame sizes. The positively charged domain was defined as being the stretch with the highest *Q* value and *Q*c ≥ 0.23. (**A**) Frequency distribution of the domain sizes retrieved by the variable frame program using the same 133 viruses analyzed in Fig. 3B as the input. The inset shows the Pearson correlation between the Total Q_max30res_ calculated using the fixed frame program and the Total Q_max*n*res_ calculated using the variable frame program. (**B**) Examples of sequences found by the variable frame (marked in red) and fixed 30 residues frame program (marked in blue).

Although the fixed-frame method is fast and straightforward, it lacks size resolution. Considering that the positively charged domain must be a region that concentrates the positive charges of the protein, we devised a method that expands the frame length possibilities and includes *Q* concentration (*Q*c = *Q*/stretch size) as an additional factor to select the positively charged domain. The new program starts from a pre-determined search frame (e.g., 8 residues) and saves the stretch with the higher net charge as *Q*_max*n*res_. Then, the search is re-initiated with 9 residues. The program will replace the previous stretch if the new stretch has a higher *Q*_*max*_ value and if the *Q*c is higher than or equal to a predetermined threshold. This approach minimizes the identification of long stretches that have an uneven distribution of the positively charged amino acids (i.e., low Qc). The program continues the search until it exhausts all the frame size possibilities, limited by the sequence size (Fig. S2). We tested the new program using the same dataset analyzed in Fig. 3B. The search parameters were the minimum frame size of 10 amino acids and *Q*c ≥ 0.23 (see next section for threshold selection criteria). The frequency distribution of the *Q*_max*n*res_ stretch sizes is shown in Fig. 4A. The majority of the identified domains had between 15 and 60 residues (median = 46). Some viruses with highly charged domains (*Q*_max30res_ ≥ 20) retrieved stretch sizes with more than 100 amino acids. In these cases, the *Q*c threshold could be increased to better capture the domain enriched in positively charged residues. Next, we compared the Total *Q*_max_ values found by the fixed and variable frame programs for each of the 133 sequences in the data set used in Fig. 3B. The strong correlation (Pearson r = 0.92; *p*-value <0.001) showed that the variable window program generally introduces discrete *Q* adjustments without distorting the data. Indeed, the variable frame outputs closely reproduced the plot from Fig. 3 (Fig. S5). Figure 4B shows examples of sequences found by the variable frame (red) and fixed 30 residues frame program (blue). In the case of MS2, the variable frame program retrieved the same domain indicated by the fixed frame program, but for CHIK, the new program correctly added an important arginine-rich region to the positively charged domain. The overlap between the sequences identified by the fixed frame and the variable frame program demonstrates that the methods are consistently retrieving the same protein regions. Therefore, we concluded that while using the variable frame could be useful to finely locate positively charged domains, the 30 amino acid residue frame is enough to capture the positively charged regions involved in genome stabilization and can be used for further analysis.

### Arginine-rich, highly positively charged domains are a hallmark of viral capsid proteins

**Figure 5:**
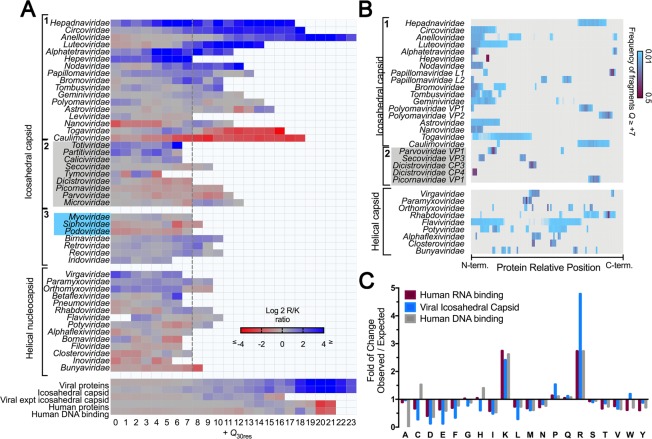
Composition and location of positively charged domains from viral capsid proteins: (**A**) The arginine and lysine residues of fragments with Q_30res_ ≥ 0 of 1,100 capsid proteins from 49 virus families were calculated. The log2 R/K ratio per net charge value is shown as a heatmap, ranging from red (K-enriched) to blue (R-enriched). Groups 1 and 2 (grey box) contain the icosahedral viruses shown in Fig. 3A; the latter corresponds to families identified as outliers. Group 3 contains bacteriophages and complex multicomponent icosahedral capsids that were not analyzed in Fig. 3. From this plot, we see that all groups included in the linear fit of Fig. 3A had at least one segment with Q_30res_ ≥ +7 (dashed line). (**B**) A heatmap indicates the frequency values of fragments with Q_30res_ ≥ +7 according to their position in the primary structure. The protein lengths were normalized and divided into bins of 0.01. (**C**) The sequence of fragments with Q_max30res_ ≥ +7 from viral capsid proteins (group 1 panel A) and human nucleic acid-binding proteins was used to determine the amino acid composition of positively charged segments. The panel shows the amino acid enrichment in relation to the total Swiss-Prot proteome amino acid composition.

Next, we examined the composition and location of these positively charged protein segments in viral capsid proteins (Fig. 5). We complemented the protein dataset analyzed in Fig. 3 (Fig. 5, groups 1 and 2) with icosahedral viruses with complex capsids (group 3) and helical virus NCPs, totaling 1,100 entries from 49 virus families. In Fig. 5A, we show the ratio between arginine and lysine according to positive values of *Q*_30res_ found in CPs from different viral families. While approximately 60% of the viral proteins have more Arg than Lys residues in their most charged segment, the human proteome follows an opposite trend (see the bottom of Fig. 5A). The bias towards arginine was stronger among the families that are included in the linear fit shown in Fig. 3A (see upper Fig. 5A, group 1), but there were important exceptions: the inliers *Togaviridae* and *Caulimoviridae* have Lys-rich segments. Among the helical viruses and the other icosahedral capsid proteins, we observed mixed patterns of R/K usage, but arginine is still preferred, especially in highly positively charged segments, such as the ones present in the histone-like proteins of adenoviruses (group 3). Another pattern that emerged from Fig. 5A is that all viruses from group 1 have at least one segment with *Q*_30res_ ≥ +7. Although this feature is not exclusive to Group 1, we used this value as a threshold to the calculation with flexible sequence frames (Fig. 4) and to map the location of positively charged segments in the primary structure of capsid proteins (Fig. 5B). To allow a direct comparison, protein lengths were normalized and split into bins of 0.01; colors indicate the frequency of fragments with *Q*_30res_ ≥ +7. Helical viruses presented a more scattered and fragmented pattern of charge distribution than the inliers, which tend to have their positively charged segments concentrated in one or both extremities of the capsid protein, usually in the N-terminus. Finally, in Fig. 5C, we analyzed the amino acid composition of the most charged segment of each virus from Group 1 *Q*_*max*30res_ and compared this with a dataset of *Q*_*max*30res_ of human nucleic acid-binding proteins. Viruses had more arginine, proline, and tryptophan than the human dataset (Fig. 5C). We looked for recurring patterns or known motifs in these sequences using MEME (data not shown)^28^. The program retrieved expected motifs for the human data sets, such as RGG and RGR motifs for the human RNA-binding proteins^29^ and zinc fingers and homeobox motifs for the DNA-binding proteins^30^. However, for the viral data set, no known nucleic-acid motifs were identified, and the few patterns retrieved by the program matched entries from the same family (not shown). This result confirms the unique structural makeup of viral capsid positively charged domains with other DNA- and RNA-binding proteins.

**Figure 6:**
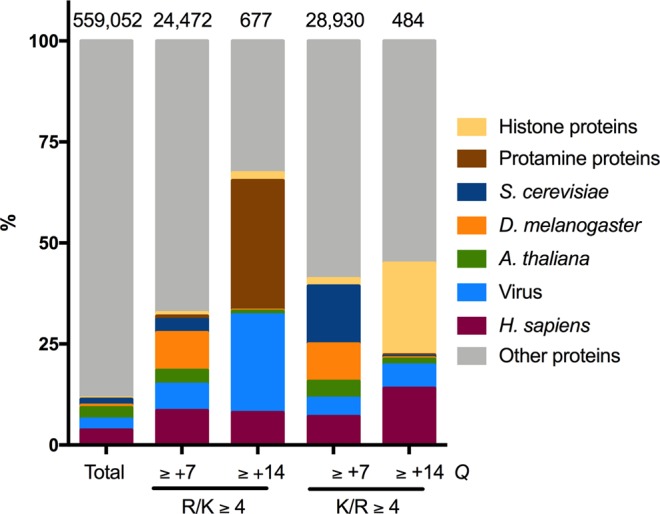
The proportion of proteins containing positively charged segments in the Swiss-Prot database. Protein sequences derived from the reviewed Swiss-Prot data-bank were used as input for a program that calculates the net charge of every consecutive 30 residue amino acid segments (*Q*_30res_). The arginine and lysine residues of fragments with *Q*_30res_ ≥ 7 or + ≥ 14 were determined. Proteins containing at least one segment with *Q*_30res_ ≥ +7 or ≥+14 with R/K ≥ 4 or K/R ≥ 4 were listed according to the organism or function.

We found that the high frequency of positively charged domains found in many viruses (Fig. 1) is due to the existence of icosahedral viral capsids (Fig. 2), an extremely specialized quaternary arrangement of proteins and nucleic acids, whose function and structure have no counterpart in cellular organisms. By analyzing the proteins by their *Q*_max30res_ and R/K values (Fig. 6), we found that only 0.1% of all proteins of the Swiss-Prot database have at least one or more stretches with *Q*_30res_ ≥ +14 and R/K ≥ + 4. Approximately 25% of these are viral capsid proteins, a striking feature of viruses, considering that they represent only 3% of the Swiss-Prot proteome (Fig. 6). Eukaryotes are the only other group with a considerable number of proteins with a similar constitution. Nevertheless, these proteins represent a tiny fraction of the individual organism’s proteome (e.g., only 55 proteins with Q_30res_ ≥ +14 and R/K ≥ 4 in 20,415 human proteins). Among these proteins, nucleic acid binding proteins and, more notably, Protamines, small proteins expressed exclusively during spermatogenesis and are involved in DNA hyper-condensation^31^ (Fig. 6). The arginine side chain possesses a guanidinium group, able to form bidentate bonds that are advantageous to maximize nucleic acid folding and packing compared to Lys^32,33^. Moreover, arginine-rich cell-penetrating peptides are more efficient than lysine-rich peptides, probably because of the bidentate interaction forces membrane curvature and destabilization^34^. Hence, arginine seems to be the optimal amino acid to condense and stabilize the viral genome and to facilitate membrane interaction. Nevertheless, unlike the negatively charged amino acids that can be found in stretches of 30 consecutive residues, the concentration of R and K in a short protein segment is limited (Fig. 1). The adverse effect of exceptionally positively charged protein segments on ribosomal synthesis efficiency may be among the selective pressures acting against repetitions of R or K in all organisms^25^. Additionally, the size and composition of positively charged viral domains might be controlled by other factors. Viral nucleic-acid structural features that are rare in host cells usually serve as molecular targets for the innate immune response^35^, and R-rich domains may function as a viral protein-specific pattern.

## Discussion

The calculation of the capsid internal net charge shown in Fig. 3 follows the most straightforward methodology published to date^12,15,24^ since the only criterion for R-arm identification was the assumption that it is the most positively charged segment of the capsid protein. From the correlation between capsid total *Q*_max30res_ and *Q*_genome_, we could distinguish at least 3 groups: complex bacteriophages that do not have a typical R-arm and pack the genome through molecular motors (blue group Fig. 3A); small viruses that deviate from the linear fit between genome and capsid charge (grey group Fig. 3B); and viruses for each the total net-charge derived from the positively charged domains strongly correlates with genome packaging capacity with Pearson r = 0.91, *p* < 0.0001 (viruses composing the linear fit Fig. 3B). This last group includes at least four RNA viruses for each the involvement of positively charged domains in genome packaging was experimentally demonstrated^4,24^. The 1/slope value that gives the capsid/genome charge ratio was 1.4. This value is generally in line with previous data indicating that (+)RNA viral capsids are overcharged, meaning that the *Q*_genome_ is not completely neutralized by protein-derived positive charges^4,10,11^.

A strong correlation does not necessarily imply causation (ie. genome size is determined by R-arms charge), but the fact that so many viruses followed approximately the same capsid/genome charge ratio suggests a common underlying pressure controlling genome size and R-arm charge. We tested whether R-arm size would correlate with the number of capsid subunits (as an approximation for capsid radius). The correlation was not significant when we considered all families (r = − 0.058, p-value = 0.510), not even when we excluded the big bacteriophages (r = − 0.158, p-value = 0.1139) (data not shown).

While our analysis implies a general role for positively charged domains in capsid assembly and genome interaction for all inlier families including some DNA viruses, it is important to note that the details of the assembly pathways can be highly diversified. Some viral capsids rely more heavily on CP-CP interactions for assembly, as suggested by the formation of empty capsids in the absence of positively charged domains (e.g., *Hepadnaviridae*^5^, while others are entirely dependent on R-arms to form the capsid (e.g., *Nodaviridae*^17^ and *Alphatetraviridae* (John E. Johnson, personal communication)). *Polyomaviridae* and *Papillomaviridae* are known to pack their genome with histones, suggesting that the R-arms are not sufficient to stabilize or condense the stiffer dsDNA^36^.

One unexpected finding among the inliers was MS2 and other *Leviviridae* bacteriophages. MS2 depends on the RNA binding protein A for genome packaging^37^ and is the prototype virus for assembly mechanisms driven by specific interaction between the capsid protein and RNA structural elements^38,39^. In fact, instead of being in a flexible arm, the most positively charged segment of the MS2 capsid was located on the internal beta-strand in close contact with the packaged RNA (Fig. S6). However, mutations in some, but not all positively charged amino acid residues of this domain interfere with the RNA packaging capacity^40^, which indicates that charge balance and neutralization plays, at most, a secondary role in *Leviviridae* assembly and stability.

The bacteriophages highlighted in blue in Fig. 3A and the 10 virus families that deviated from the linear fit represent assembly strategies with even less significant contribution of electrostatic interactions coming from a positively charged CP domain. In all cases, the genome charge exceeded the expected internal capsid charge (Fig. 3A). Counter ions, such as Mg^2+^, and other positively charged molecules, such as polyamines, have been found inside bacteriophages and eukaryotic viral particles and may help to balance charges in these viruses^41,42^.

Next, we carefully examine these outlier families (two dsDNA, 6 +RNA, and 1 ssDNA viral families) and discuss how their position in Fig. 3 can help to understand the functional aspects of the capsid.

The dsRNA families *Totiviridae* and *Partitiviridae* share similar simple capsid architecture, with 60 CP dimers forming a T1 capsid. All dsRNA viruses, including the more complex reoviruses and birnaviruses, replicate their genome and transcribe their mRNA inside an assembled capsid that also encloses the RNA-dependent RNA polymerase. More than transporting the genome, these particles are part of the viral factory, preventing the detection of viral dsRNA species by cellular proteins^43^. Because these capsids must sustain variable levels of RNA content during viral replication, it is reasonable that these families diverged from the group belonging to the linear fit. Among the ssRNA outliers, we found *Caliciviridae*, the 3 families of picornavirales present in the dataset (*Dicistroviridae*, *Secoviridae*, and *Picornaviridae*); and *Tymoviridae*. A recent sequence-similarity network analysis of single jelly-roll capsid proteins from RNA viruses revealed two large clusters, one containing most of the ssRNA viruses present in our data set and another formed by picornavirales and *Caliciviridae*^44^. Although the capsid architecture is not the same, both groups pack VPg, a small protein bound to the genome 5′−end^45^. Picornaviruses form pseudo-T = 3 capsids containing 4 different proteins. Segments with charge > +7 were found in few entries and were restricted to one or two CPs. The primary role of these domains is unknown, but they may participate in membrane interaction, as already demonstrated for dicistroviruses CP4^46^. Most viruses from *Caliciviridae* assemble their capsid with one type of CP arranged in 90 dimers in a T = 3 lattice. No segments with *Q* ≥ +7 were found in *Caliciviridae* CPs. Our data reinforce the structural similarities between these two groups and suggest a common yet unknown mechanism for genome stabilization and assembly. The *Tymoviridae* capsid proteins are also devoid of segments with Q ≥ +7. An X-ray structural model of DYMV includes densities corresponding to ordered RNA inside the capsid, but no positively charged residues are present in the interaction interface^47^ The *Parvoviridae* and the *Microviridae* phages were the only T = 1 ssDNA viruses identified as an outlier family. These viruses enclose the largest genomes among the eukaryotic ssDNA viruses (~ 5 kb) but have charge values similar to the tiny *Nanoviridae* (~1 kb). *Parvoviruses* present 3 variations of the cap gene product, all having an overlapping amino acid sequence with similar C-termini. The most charged segment is a short Lys-enriched region unique to VP1. Because this CP variant is the least abundant, our charge calculation is probably overestimated. The capsid is primarily formed by VP2 proteins that have a very conserved ssDNA binding pocket^48^. The binding site shows an ordered loop of 9 nucleotides that coordinates two Mg^2+^. This stable and structured contact between the genome and the protein shell may represent an alternative strategy to the long super-charged R-arms that are observed in circovirus and anellovirus^6,48^. Instead of having a CP containing a positively charged domain, viruses from the *Microviridae* family have two short (<40 residues) R-rich proteins H and J that together account for most of the capsid charge. Both are thought to escort and direct the genome towards the interior of a pre-formed capsid in a packaging process that is coupled to ssDNA synthesis^1,27^. It should be noted that there is a considerable degree of uncertainty regarding the stoichiometry of these small peptides, especially for H protein^1,27^, which can explain the observed wide variation in the Total *Q*_max30res_ for similar genomes sizes (Fig. 3).

Despite the simplicity of the calculation, we reproduced the general findings obtained with some (+)RNA viruses and showed that the striking correlation between genome charge and positively charged protein domains could be extended to other viruses with different genome types (approximately 67% of the eukaryotic icosahedral virus families analyzed in Fig. 3B). However, the outlier position of *Microviridae* family that depends on positively charged proteins for genome packaging, and the inlier position of *Leviviridae* that are known to use alternative strategies to drive assembly, demonstrated that our plot cannot provide binary answers concerning the involvement of R/K rich proteins in capsid formation. Moreover, we do not rule out that these R-arms assist capsid assembly in other ways besides (or alternative) to the genome charge neutralization^49^. Moreover, viral proteins are notably versatile and more than one functional pressure might be shaping the final composition and charge of R-arms. For example, positively charged domains of polyomavirus are known to drive endosome scape^20^; and R-rich segments of *Tetraviridae* function as lytic peptides^16^. All these viruses are inliers and these additional roles are not necessarily excluding the R-arm function in charge balance/neutralization.

## Methods

### Data sources

The protein database Swiss-Prot at Uniprot.org was used as our source of primary protein sequences. Protein function, taxonomic, and structural information were retrieved from Uniprot.org, Viralzone, and Viperdb. Genome sizes for all viruses were obtained at the National Center for Biotechnology Information (NCBI) database. Reference sequences were used when available. UniRef advanced search options were used to retrieve datasets according to organism or protein function. The advanced search options (UniProt:(proteome:(taxonomy:“Viruses [10239]”) reviewed:yes) AND identity:1.0). For viral capsid proteins, we used the advanced search options (proteome:(taxonomy:“Viruses [10239]”) goa:(“viral capsid [19028]”) AND reviewed:yes) followed by a manual selection of major capsid proteins. Advanced search options for H. sapiens (reviewed:yes AND organism:“Homo sapiens (Human) [9606]”), D. melanogaster (reviewed:yes AND organism:“Drosophila melanogaster (Fruit fly) [7227]”) and A. thaliana (reviewed:yes AND organism:“Arabidopsis thaliana (Mouse-ear cress) [3702]”) were used as a source for the proteomes. For protein class analyses, proteins were further separated by their Gene Ontology ID (GO:3677 DNA binding; GO:3723 RNA binding; GO:34061 DNA polymerase activity; GO:34062 5′-3′ RNA polymerase activity; GO:19031 viral envelope). Viral capsid proteins were separated in helical or non-helical regions by helical viral capsid Gene Ontology ID (GO:19029).

### Net charge calculation

We used a program that screens the primary sequence of a given protein and calculates the net charge in consecutive frames of a predetermined number of amino acids (10, 30, or 60 were used). For the net charge determination, K and R were considered +1; D and E were considered -1; every other residue was considered 0. The N and C termini charges were disregarded. In a previous publication, we have shown that these simplified parameters generated similar results to a calculation using partial charges of individual amino acids at pH 7.4 according to their p*K*a values and the Henderson-Hasselbach equation^25^.

Our algorithm uses, as input, a fasta file containing the amino acid sequence of multiple proteins (see Data sources section). The algorithm initially establishes a stretch containing a predetermined number of amino acids (#1 to #30, for example). The stretch charge is calculated, and the charge value and the position of the first amino acid are temporarily saved to the memory. Then, our algorithm advances to the next stretch, from amino acid #2 to amino acid #31, and performs the same analysis. The algorithm continues advancing through the protein until it reaches the stretch between the amino acids N-29 and N, where N is the total amount of amino acids in that protein. If its charge is higher than the charge previously saved in the memory, the current values of the charge and the position are replaced there.

A second algorithm was developed to compare the net charge of stretches with different sizes. Initially, this algorithm works as the first algorithm, but when it finishes the search for the highest charge, it restarts the same protein, analyzing stretches of the pre-determined value +1 amino acids. This process is repeated until all possible sizes are calculated. If a stretch has a higher charge than any of the previously analyzed, then the value, its position, and its size (in the number of amino acids) are replaced in the memory. Since this program compares the charge of stretches of different sizes, we established that a long stretch would only be considered to have a higher charge if it has a higher charge ratio (charge divided by the stretch length) than the previously saved in the memory (see Supplemental Fig. S2 for a graphic representation).

Overrepresentation of net charge values varying from −30 to +30 calculated in 30 width amino acid stretches was performed by right-sided P[X ≥ x] hypergeometric tests. For the enrichment analysis of any specific net charge value, hypergeometric distributions were used for sampling without replacement. The density of these distributions with parameters *m*, *n* and *k* were given by Equation 1:

1 $$p(x)=\frac{m}{n}\times \frac{n}{k-x}/\frac{m+n}{k},\forall x=0,\ldots ,k,$$

where *x* was the number (−1) of amino acid stretches in proteins of any specific species (e.g.*, D. melanogaster, A. thaliana, H. sapiens*) or categories (e.g., viral particle, polymerase, nucleic acid binding) with any specific net charge; *m* were the number of amino acid stretches in all Swiss-Prot database proteins with any specific net charge; *n* was the number of all Swiss-Prot database of any net charge but the one being analyzed; *k* was the number of amino acid stretches of any charge in proteins of any species or categories being analyzed. Accordingly, the expected number of amino acid stretches in proteins of any specific species or categories with any net charge value were calculated by *m* × [*k*/(*m*+*n*)]. False discovery rate (FDR) controlling approach was used to reduce the type I error from performing multiple comparisons.

### Identification of proteins with positive net charge and determination of lysine to arginine ratio

To study the identity of proteins with positive net charge stretches enriched on arginine’s (R) or lysine’s (K), we designed and implemented another algorithm that calculated the net charge of amino acids at a determined ratio of R/K or K/R. The calculations of net charge, total arginine, total lysine, and the ratio among R/K or K/R were calculated by Equation 2:

2 $$Jj=\frac{{\sum }_{i=0}^{n}Rij}{{\sum }_{i=0}^{n}Kij},$$

where *Jj* is the ratio R/K for the respective *j* net charge, *Rij* is the number of arginines found in stretch *i*, *Kij* is the number of lysines found in stretch *i*, and n is the total number of stretches found.

Statistical analyses and graphical displays were made either with R version 3.5.2 or Graph-pad Prism 7.0 software. The details of *in silico* implementation and the code software were posted at https://github.com.

**Table 1:** Software and algorithms.

Software and Algorithms	Source	Identifier
Calculation of net charge	Requião *et al*., 2017	
Calculation of R/K ratio	This paper	https://github.com/mhoyerm/Total_ratio
Identify proteins of determined net charge and R/K ratio	This paper	https://github.com/mhoyerm/Modulate_RK
Identify proteins of determined net charge and K/R ratio	This paper	https://github.com/mhoyerm/Modulate_KR

## Supplementary information

Supplementary figures.

Supplementary File 3.

Supplementary File 4.

## Data Availability

The algorithms used in this work are available in the GitHub repository (Table 1).
