# Supplementary material for: Viruses with different genome types adopt a similar strategy to pack nucleic acids based on positively charged protein domains
Source: Sci Rep. 2020 Mar 25;10:5470. doi: 10.1038/s41598-020-62328-w (PMC7096446; doi:10.1038/s41598-020-62328-w)
Supplement: Supplementary file 1 — Supplementary figures. [file 41598_2020_62328_MOESM1_ESM.pdf]

Viruses with different genome types adopt a similar strategy to pack nucleic acids based on positively charged protein domains

Rodrigo D. Requião[1], Rodolfo L. Carneiro[1], Mariana Hoyer Moreira[1], Marcelo Ribeiro-Alves[2], Silvana Rossetto[3], Fernando L. Palhano*[1], and Tatiana Domitrovic*[4]

[1] Universidade Federal do Rio de Janeiro, Instituto de Bioquímica Médica Leopoldo de Meis, Rio de Janeiro, 21941-902, Brazil
[2] Fundação Oswaldo Cruz, Instituto Nacional de Infectologia Evandro Chagas, Rio de Janeiro, 21040-900, Brazil
[3] Universidade Federal do Rio de Janeiro, Instituto de Matemática, Rio de Janeiro, 21941-902, Brazil
[4] Universidade Federal do Rio de Janeiro, Instituto de Microbiologia Paulo de Góes, Rio de Janeiro, 21941-902, Brazil

* To whom correspondence should be addressed. Tel: +55 21 55 3938-6761; Email: domitrovic@micro.ufrj.br. Correspondence may also be addressed to palhano@bioqmed.ufrj.br.

A

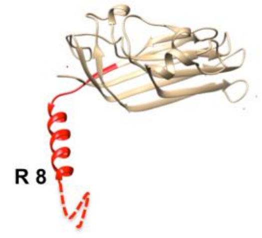

>sp|P03606|CAPSD_STNV1 Capsid protein OS=**Satellite tobacco necrosis virus**

MA**K**QQNN**RRK**SATM**R**AV**KR**MINTHLEH**KR**
FALINSGNTNATAGTVQNLSNGIIQGDDINQR
SGDQVRIVSHKLHVRGTAITVSQTFRFIWFR
DNMNRGTTPTVLEVLNTANFMSQYNPITLQ
QKRFTILKDVTLNCSLTGESIKDRIINLPGQLV
NYNGATAVAASNGPGAIFMLQIGDSLVGLWD
SSYEAVYTDA

B

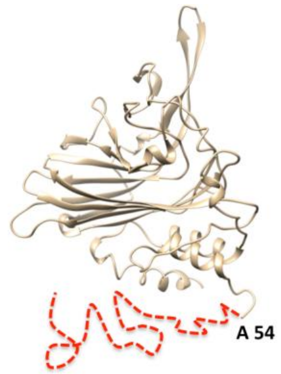

>sp|P12870|CAPSD_FHV Capsid protein alpha OS=**Flock house virus**

MVNNN**R**P**RR**Q**R**AQ**R**VVVTTTQTAPVPQQNVP**R**NG**RRR**
RN**R**T**RR**N**RRR**V**R**GMNMAALTRLSQPGLAFLKCAFAPPD
FNTDPGKGIPDRFEGKVVSRKDVLNQSISFTAGQDTFILI
APTPGVAYWSASVPAGTFPTSATTFNPVNYPGFTSMFGT
TSTSRSDQVSSFRYASMNVGIYPTSNLMQFAGSITVWKC
PVKLSTVQFPVATDPATSSLVHTLVGLDGVLAVGPDNFSE
SFIGVFSQSACNEPDFEFNDILEGIQTLPPANVSLGSTGQ
PFTMDSGAEATSGVVGWGNMDTIVIRVSAPEGAVNSAIL
KAWSCIEYRPNPNAMLYQFGHDSPPLDEVALQEYRTVA
RSLPVAVIAAQNASMWERVKSIIKSSLAAASNIPGPIGVAA
SGISGLSALFEGFGF

C

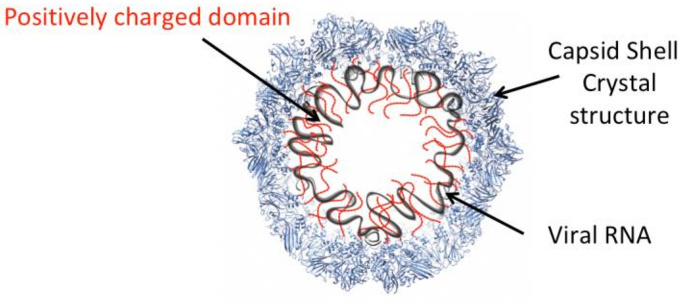

S1. Examples of positively charged domains of virus capsid proteins. Protein segments rich in arginine and/or lysine from the Satellite Tobacco Necrosis Virus STNV (A) and Flock House virus FHV (B) are highlighted in red the capsid protein crystal structure (pdb coordinates 4bcu and 6f2s, respectively) and protein sequence (uniprot). The positively charged domain is usually within a helical region, as in STNV, or within a flexible segment of the protein that is not resolved in the crystal structure (represented by red strings). The X-ray tridimensional coordinates of FHV capsid were used to generate a cut-away view of the T=3 icosahedral capsid. Short patches of RNA interact with the capsid protein and are ordered, but the vast majority is dynamic (represented by black lines) and is probably intertwined with the Arg-rich protein domain.

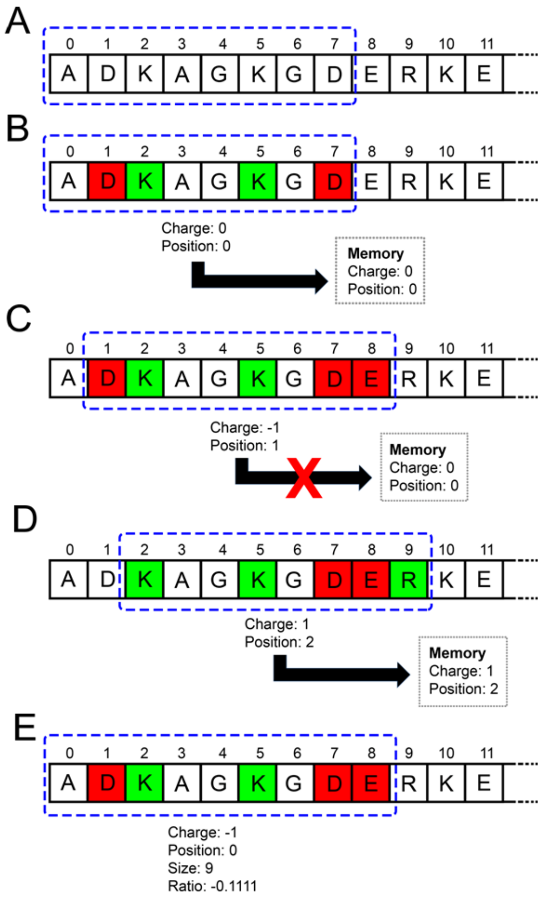

S2. Net charge calculation. Our algorithm uses, as input, a fasta file containing the amino acid sequence of multiple proteins (see Data sources section). A. Initially, it establishes a stretch containing a predetermined number of amino acids. In our study, we initially used 10, 30, and 60 amino acids frames, but, for the sake of simplicity, we demonstrate it here with 8 amino acids: from amino acid #0 to amino acid #7. The analyzed stretch is highlighted as a dashed blue rectangle. B. The net-charge is then calculated. For each Lysine (K) or Arginine (K), it is counted +1, and for each Aspartic Acid (D) or Glutamic Acid (E), it is counted -1. The charge value and the position of the first amino acid are temporarily saved to the memory. C. and D. Then, our algorithm advances to the next stretch, from amino acid #1 to amino acid #8. It keeps advancing through the protein until it reaches the stretch between the amino acids N-7 and N, N being the total amount of amino acids in that protein. If a charge value is higher than the one previously saved in the memory, the current value and position are replaced. E. A second algorithm expands the possibilities. When it finishes the search for the highest charge in a frame of 8 amino acids in a protein, it restarts the same process but with 9 amino acid frame. This is repeated until all possible sizes are calculated. If a stretch has a higher charge than any of the previously analyzed, then the value, position and size (in number of amino acids) are replaced in the memory. Since this program compares the charge of stretches of different sizes, we established that a longer stretch will only replace a shorter one if it has a charge concentration (charge divided by the stretch length) higher than a predetermined threshold previously saved in the memory.

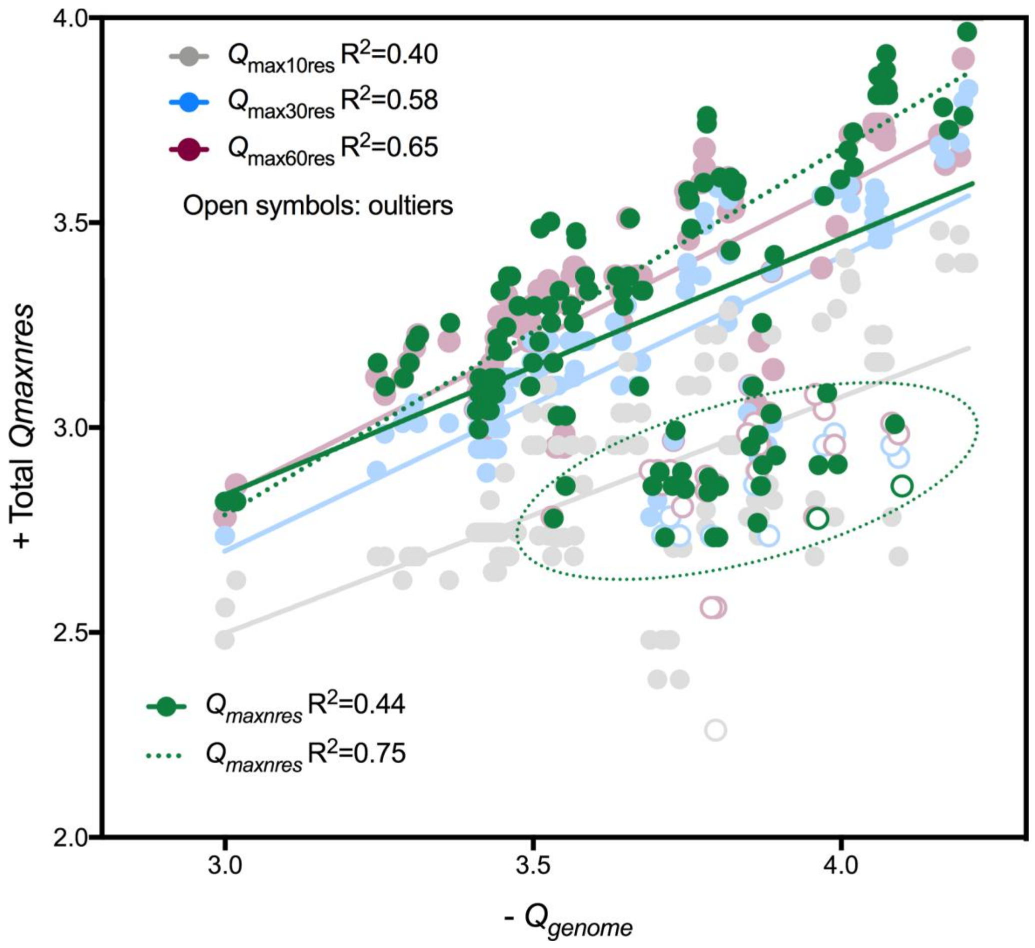

S5. Correlation between genome size and capsid charge calculated by different R-arm sizes. The maximum net-charge value found in 10, 30 or 60 or variable amino acid residues frames was multiplied by the number of subunits forming the capsid (Total Q_{max}) of 129 viruses from 25 different families. The total nucleic-acid net charge was calculated from the number of nucleotides residues in the genome (Q_{genome}). For multipartide viruses, the longest genome segment was considered for the plot. A straight line fit (Q = 5 %) for the entire dataset was calculated and the open symbols indicates the outliers. Dashed fit represents the linear regression calculates after removal of the viruses identified as outliers (dashed circles).

>P03612 CP *Escherichia* phage MS2 Leviviridae
MASNFTQFVLVDNGGTGDVTVAPSNFANGVAEWISSNSRSQAYKVTCSVRQS
SAQNRKYTIKVEVPKVATQTVGGVELPVAAWRSYLNMELTIPIFATNSDCELIVK
AMQGLLKDGNPIPSAIAANSGIY

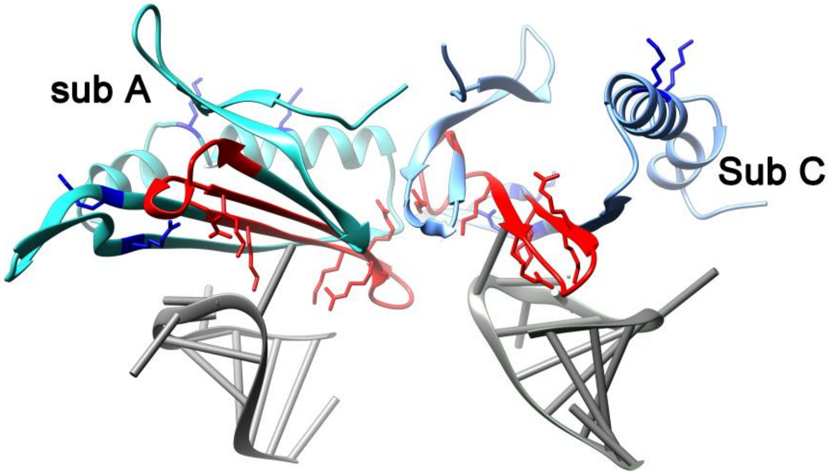

S6. *Leviviridae* positively charged domain is embedded within an ordered region of the capsid in close contact with DNA. Panel A shows the positively charged domain identified by the fixed and variable window of the program. Panel B: X-ray tridimensional coordinates of MS2 capsid (pdb: 5msf) showing two subunits of capsid protein and a fragment of ordered nucleic acid (grey). Positively charged domains are shown in red.
